# Comments on “Treatment of Pericardial Effusion Through Subxiphoid Tube Pericardiostomy and Computerized Tomography - Or Echocardiography - Guided Percutaneous Catheter Drainage Methods”

**DOI:** 10.21470/1678-9741-2019-0331

**Published:** 2019

**Authors:** Kyriakos Spiliopoulos, Dimitrios Magouliotis, John Skoularigis, Filippos Triposkiadis

**Affiliations:** 1 Department of Thoracic and Cardiovascular Surgery, Larissa University Hospital, Larissa, Greece.; 2 Department of Surgery, Larissa University Hospital, Larissa, Greece.; 3 Cardiology Department, Larissa University Hospital, Larissa, Greece.

Dear Editor,

We read with great interest the article by Colak et al.^[1]^ “Treatment of Pericardial Effusion Through Subxiphoid Tube Pericardiostomy and Computerized Tomography - or Echocardiography - Guided Percutaneous Catheter Drainage Methods” published recently in the Brazilian Journal of Cardiovascular Surgery^[1]^. The issue is very relevant as long as the diagnosis and treatment of pericardial effusion and subsequently cardiac tamponade have evolved over the years with a tendency towards a more comprehensive diagnostic workup and less traumatic intervention. We would like to take the chance to add some thoughts about the treatment and especially the surgical management of the entity.

The series of Colak et al.^[1]^ consists of 553 patients treated due to pericardial effusions during a time period of 14 years in their center. This study population represents one of the biggest, if not the largest, reported single center studies dealing with the issue. Regarding the treatment, three approaches were applied, with the majority of the cases (n: 480) being treated by subxiphoidal tube pericardiostomy, and a relatively small amount of patients (n: 73) through a percutaneous catheter drainage.

In general, in about 60% of cases, the effusion is resulted from a known disease, thus therapy should be targeted at the etiology as much as possible. In order to provide the most effective treatment, several algorithms for triage and management have been proposed^[2,3]^.

However, in the presented study it is not clear if the authors followed an algorithm, resulting probably in the abovementioned unequal distribution (in favor of subxiphoidal pericardiostomy) of the applied treatment approaches. The surgical approach, through a subxiphoidal incision as described by the authors, remains the gold standard for pericardial drainage and biopsy. Nevertheless, we would like to advocate in this context for the left lateral minithoracotomy incision as the approach of choice, especially in cases where the effusion recurrence rate, due to the underlying disease, is expected to be high. According to our institutional experience of more than 100 cases drained with this approach (data not published), the thoracotomy incision enables, on the one hand, the establishment of a classical pericardiopleural window, and on the other hand, it makes feasible a more extensive pericardiectomy compared to the subxiphoidal approach. In addition, the procedure can be performed under conditions with local anesthesia, and in our eyes, it seems to be accompanied by less complications than the subxiphoidal pericardiostomy.
